# Triphenyltin Chloride Delays Leydig Cell Maturation During Puberty in Rats

**DOI:** 10.3389/fphar.2018.00833

**Published:** 2018-08-10

**Authors:** Linchao Li, Lubin Xie, Leikai Ma, Yong Chen, Xianwu Chen, Fei Ge, Tongliang Huang, Lanlan Chen, Tingting Hong, Xiaofang Chen, Qiqi Zhu, Xingwang Li, Ren-Shan Ge

**Affiliations:** ^1^Department of Anesthesiology, The Second Affiliated Hospital and Yuying Children’s Hospital, Wenzhou Medical University, Wenzhou, China; ^2^Department of Obstetrics and Gynecology, The Second Affiliated Hospital and Yuying Children’s Hospital, Wenzhou Medical University, Wenzhou, China

**Keywords:** reproductive toxicity, triphenyltin, Leydig cells, kinase, development, ROS, apoptosis, rats

## Abstract

Triphenyltin chloride (TPT) is present in a wide range of human foods. TPT could disrupt testis function as a potential endocrine disruptor of Leydig cells. However, the effect of TPT on pubertal Leydig cell development is still unclear. The objective of the current study was to explore whether exposure to TPT affected Leydig cell developmental process and to clarify the underlying mechanisms. Male Sprague-Dawley rats at 35 days of age were randomly divided into four groups and received normal corn oil (control), 0.5, 1, or 2 mg/kg/day TPT for 18 days. Immature Leydig cells isolated from 35-day-old rat testes were treated with TPT (10 and 100 nM) for 24 h *in vitro*. *In vivo* exposure to ≥0.5 mg/kg TPT lowered serum testosterone levels and lowered *Star* mRNA. TPT at 2 mg/kg also lowered *Lhcgr*, *Cyp11a1*, *Hsd3b1*, *Hsd17b3* as well as pAKT1/AKT1, pAKT2/AKT2, and pERK1/2/ERK1/2 ratios. *In vitro* exposure to TPT (100 nM) increased ROS production and induced cell apoptosis rate in rat immature Leydig cells. In conclusion, TPT exposure disrupts Leydig cell development possibly via interfering with the phosphorylation of AKT1, AKT2, and ERK1/2 kinases.

## Introduction

Organotins are one of the classes of chemicals as endocrine disruptors. Among them, triphenyltin chloride (TPT) has been intensively used in industry and agriculture, as plastic stabilizers, pesticides, and marine antifouling paints. Concerns about environmental pollution of TPT have arisen due to its high affinity for particulate matter and its tendency to be enriched in the sediments (de Araujo et al., 2018). TPT has been detected in blood samples of Finnish people and TPT levels in fishermen were higher because they ate more sea-foods (Rantakokko et al., 2008).

An earlier study found that TPT could effectively cause infertility in both male and female flies (Kenaga, 1965). This promoted researchers to raise the possibility that TPT might also affect the reproduction of mammals. Researchers from Battelle Research Laboratories conducted a dose (1, 2, 3, 6, and 12 mg/kg body weight/day) range-finding study for TPT toxicity on male reproduction in rats (Battelle, 1981) and found that TPT at a dose of 12 mg/kg caused the reduced fertility in male rats. Although the exact mechanism of TPT toxicity on male reproductive system is not clear, *in vitro* studies indicated that the Leydig cells could be the target of TPT. In an *in* *vitro* study using isolated pig Leydig cells, TPT was found to be a direct inhibitor of 17β-hydroxysteroid dehydrogenase 3 (HSD17B3, an *Hsd17b3* product), an enzyme catalyzing the last-step testosterone biosynthesis from androstenedione, with a half maximal inhibitory concentration (IC_50_) of 48 nM, therefore inhibiting androgen production (Ohno et al., 2005). TPT was also a moderate inhibitor of P450 reductase with IC_50_ value of 22.8 μM (Ohno et al., 2005), thus suppressing 17α-hydroxylase/17-20lyase (CYP17A1, a *Cyp17a1* product), which uses P450 reductase as its electron carrier. However, whether TPT disrupts Leydig cell development during puberty is unclear.

Leydig cells existing in the interstitial compartment of the testis are unique endocrine cells, producing almost 95–99% of circulatory testosterone amount (Teerds and Rijntjes, 2007). Androgen production relies not only on the capacity of steroidogenesis of the Leydig cell *per se*, but also on the number of Leydig cells (Ye et al., 2017). In the rat, Leydig cell development during puberty is conceptually defined into four stages, which starts with the appearance of progenitor Leydig cells at 21 days postpartum from the commitment of stem Leydig cells, undergoes transition through immature Leydig cells at 35 days postpartum, and finishes the maturation into adult Leydig cells at 49–52 days postpartum (Ye et al., 2017). The late-stage maturation of Leydig cells from immature into adult cells not only has the increased capacity of pituitary luteinizing hormone (LH)-stimulated androgen production but also has the upregulated expression of steroidogenic proteins, such as LH receptor (LHCGR, an *Lhcgr* product) (Chen et al., 2017), cholesterol-transporter high-density lipoprotein receptor (SCARB1, a *Scarb1* product) and steroidogenic acute regulatory protein (STAR, a *Star* product), cholesterol side chain cleavage enzyme (CYP11A1, a *Cyp11a1* product), 3β-hydroxysteroid dehydrogenase 1 (HSD3B1, an *Hsd3b1* product), *Cyp17a1*, and *Hsd17b3* (Ge et al., 2005; Stanley et al., 2011). This maturation changes the primary androgen from 5α-androstanediol into testosterone in adult Leydig cells (Ge and Hardy, 1998). Interestingly, this late-stage maturation has 11β-hydroxysteroid dehydrogenase 1 (HSD11B1, an *Hsd11b1* product) expression being eightfold increase (Ge et al., 2005), serving a good biomarker for maturation of Leydig cells.

Since the effects of TPT on pubertal development of Leydig cells and underlying mechanism is not clear, in the current study, we exposed male rats to different doses of TPT from 35 days to 52 days postpartum and then observed the impairment of Leydig cell developmental process.

## Materials and Methods

### Chemicals and Animals

Triphenyltin chloride (purity 96.0%) was purchased from J&K Scientific (Beijing, China). Immulite2000 Total Testosterone Kit was obtained from Sinopharm Group Medical Supply Chain Services (Hangzhou, China). Trizol kit was purchased from Invitrogen (Carlsbad, CA, United States). Reverse transcriptase kit was purchased from Promega (Madison, WI, United States). Quantitative PCR (qPCR) reagent kit was purchased from Takara (Otsu, Japan). The primer information for qPCR was listed in **Supplementary Table S1**. The antibody information for Western blot and immunohistochemical staining was listed in **Supplementary Table S2**. All other reagents were obtained from Sigma-Aldrich (St. Louis, MO, United States). Male Sprague-Dawley rats (28 days of age) were purchased from Shanghai Animal Center (Shanghai, China). All animal studies were conducted according to the research protocol approved by Wenzhou Medical University Institutional Animal Care and Use Committee and were performed in accordance with the Guide for the Care and Use of Laboratory Animals.

### Animal Administration

Male rats, aged 28 days, were raised in a 12 h dark/light cycle temperature at 23 ± 2°C with relative humidity of 45–55%. Water and food were provided *ad libitum*. After 1 week of adjustment in the new environment. Animals were randomly divided into four groups (seven rats per group): 0 (corn oil, as control), 0.5, 1, and 2 mg/kg body weight/day TPT groups, respectively. TPT was dissolved in corn oil and was gavaged daily to rats starting on days 35 postpartum. Body weight of each rat was recorded daily. Rats were sacrificed on days 52 postpartum by asphyxiation with CO_2_. Trunk blood was collected and sera were prepared for the measurement of hormones [testosterone, LH, and follicle-stimulating hormone (FSH)]. Testes were taken out and weighed. One testis each animal was frozen in the liquid nitrogen for the measurement of testicular mRNA and protein levels. The contralateral testis was fixed in Bouin’s solution for the immunohistochemical analysis.

### Measurement of Serum Testosterone Concentration

Serum testosterone concentration was measured using Immulite2000 Total Testosterone Kit according to the according to the manufacturer’s instruction. The minimal detection limit of testosterone was 0.1 ng/ml. The intra-assay and inter-assay coefficients of variation were within 10%.

### ELISA for Serum LH and FSH Levels

Serum levels of LH and FSH were measured with ELISA kits according to the manufacturer’s instruction (Chemicon, CA, United States) as described (Wu et al., 2017). Briefly, serum sample was added to the pre-coated well and incubated with peroxidase-conjugated IgG anti-LH or anti-FSH for 2 h. Then, substrate solution was added and the enzyme reaction results were measured by a microplate reader at 550 nm with correction wavelength at 450 nm.

### Immunohistochemistry

One testis each rat was used for immunohistochemical staining (Vector Laboratories, Inc., Burlingame, CA, United States) according to the manufacturer’s instructions. Testis blocks were prepared according to a stereological method as previously described (Akingbemi et al., 2004). Six testes per group were randomly selected and cut into eight disks with each disk being cut to two pieces and one piece each testis was randomly selected and dehydrated in ethanol and xylene and then embedded in paraffin in a tissue array. Six micrometer-thick transverse sections were cut and mounted on glass slides. Approximately 10 sections were used. Avidin-biotin immunohistochemical staining for CYP11A1 (the general a marker for all Leydig cells), HSD11B1 (a specific biomarker for Leydig cells at the advanced stage) or SOX9 (a biomarker for all Sertoli cells) was conducted following manufacturer’s instructions. Antigen retrieval was done by heating at 100°C in 10 mM (pH 6.0) citrate buffer for 10 min. H_2_O_2_ (0.5%) of methanol was incubated with sections for 30 min to block the endogenous peroxidase. Sections were incubated with the CYP11A1, HSD11B1, or SOX9 polyclonal antibodies (diluted 1:200) for 1 h at room temperature. Diaminobenzidine was used for visualizing the antibody-antigen complexes, positively labeling Leydig cells by a brown cytoplasmic staining or labeling Sertoli cells by a brown nuclear staining. Mayer hematoxylin was applied in the counterstaining. The sections were dehydrated in graded concentrations of alcohol and cover-slipped with resin (Thermo Fisher Scientific, Waltham, United Kingdom). Non-immune rabbit IgG was used in the incubation of negative control sections with working.

### Counting Leydig and Sertoli Cell Number by Stereological Method

To count CYP11A1 or HSD11B1-positive Leydig cells or SOX9-positive Sertoli cells, sampling of the testis was performed according to a fractionator method as previously described (Akingbemi et al., 2004). About 10 testis sections per rat were sampled from each testis. The histochemical staining was performed as above. The total number of Leydig or Sertoli cells was calculated by multiplying the number of Leydig or Sertoli cells counted in a known fraction of the testis by the inverse of the sampling probability.

### Computer-Assisted Image Analysis of Leydig Cell Size and Nuclear Size

Leydig cells were identified by staining HSD11B1 as above. The Leydig cell size, nuclear size, and cytoplasmic size were calculated as previously described (Liu et al., 2016). Six randomly selected fields in each of three non-adjacent sections per testis were captured using a BX53 Olympus microscope (Tokyo, Japan) equipped with a digital camera interfaced to a computer. The images that were displayed on the monitor represented partial area of a testis. Cell size and nuclear size were estimated using the image analysis software (Image-Pro Plus; Media Cybernetics, Silver Spring, MD, United States). More than 50 Leydig cells were evaluated in each testis. The cell size and nuclear size were recorded as μm^3^ and cytoplasmic size was calculated by cell size minus nuclear size.

### Semi-Quantitative Measurement of CYP11A1, HSD11B1, and SOX9

CYP11A1 and HSD11B1 are the proteins of Leydig cells and SOX9 is the Sertoli cell protein. CYP11A1, HSD11B1, and SOX9 protein levels were measured using semi-quantitative measurement of the density for cell *per se* as previously described (Liu et al., 2016). Immunohistochemical stainings of CYP11A1, HSD11B1, and SOX9 were performed as above. Target protein density and background area density were measured using the image analysis software. More than 50 Leydig cells were evaluated in each testis and the protein density of each sample was averaged.

### Real-Time PCR (qPCR)

Total RNAs were purified from testes using a TRIzol solution according to the manufacturer’s instructions (Invitrogen, CA, United States). The concentration of total RNA was measured by reading the OD value at 260 nm using NanoDrop 2000. RNA integrity was assessed in a randomly chosen subset of samples using agarose gel electrophoresis, and the OD ratio of 28S to 18S rRNA was consistently greater than 1 for each sample checked, indicating high quality. Then, cDNA was synthesized using a Reverse-Transcriptase Reagents Kit according to the manufacturer’s instructions (Invitrogen, CA, United States). A SYBR Green qPCR Kit (Takara, Otsu, Japan) was applied to amplify the transcript and to analyze the gene expression levels of *Lhcgr*, *Scarb1*, *Star*, *Cyp11a1*, *Hsd3b1*, *Cyp17a1*, *Hsd17b3*, *Hsd11b1*, *Nr5a1*, *Fshr*, *Dhh*, and *Sox9* in the testis. The reaction mixture had 10 μl SYBR Green Mix, 1.6 μl forward and reverse primer mix, 1 μg diluted cDNA sample and 5–8 μl RNase-free water. The reaction process was set as follows: 95°C for 2 min, followed by 40 cycles of 95°C for 10 s, and 59°C for 30 s. The Ct value was read out and the expression level of the target mRNA was calculated using a standard method as previously described (Lin et al., 2008). The relative expression of genes was normalized to *Rps16*, the house-keeping gene, as the control. Melting curve was examined for the quality of PCR amplification for each sample. The primers were listed in **Supplementary Table S1**.

### Western Blotting

Testes were homogenized at the ice-cold PBS buffer and then were lysed with radio immunoprecipitation assay buffer (Bocai Biotechnology, China) to obtain protein samples. The protein concentrations in the supernatants were measured using the BCA Assay Kit (Takara, Japan) according to the manufacturer’s instructions. Sample proteins (30 μg) were subjected to the electrophoresis separation in 10% polyacrylamide gel containing sodium dodecyl and then the separated proteins were transferred into a polyvinylidene fluoride membrane. After being blocked with 5% free-fat milk in Tween 20-containing Tris-buffered saline for 2 h, the membrane was incubated overnight at 4°C with primary antibody against the following antigens: LHCGR, SCARB1, CYP11A1, HSD3B1, CYP17A1, pAKT1, AKT1, pAKT2, AKT2, pERK1/2, ERK1/2, and ACTIN (Antibody information was listed in **Supplementary Table S2**), respectively. The membrane was then washed and incubated with HRP-conjugated anti-rabbit or anti-goat IgG secondary antibody (1:2000, Bioword, United States) for 2 h at room temperature and washed three times. The protein bands were visualized with an enhanced chemiluminescence kit (Pierce Chemical Co., Rockford, IL, United States). The intensity of proteins was quantified using Image J software and adjusted to the ACTIN, the house-keeping protein.

### Isolation of Immature Leydig Cells

Male rats (35 days of age) were used for isolation of immature Leydig cells. The rats were sacrificed by asphyxiation with CO_2_. Testes were removed and digested with collagenase as well as Leydig cells were purified as described previously (Shan et al., 1993). In brief, the removed testes were perfused with a M199 solution containing 0.1 mg/ml collagenase via the testicular artery, digested with a mixture of 0.25 mg/ml collagenase and 0.25 mg/ml DNase for 15 min, filtered through two-layer 100-μm nylon mesh, and the cells were separated under Percoll gradient centrifugation. The cells with density of 1.070–1.088 g/ml were collected and washed. Purities of immature Leydig cell fractions were evaluated by histochemical staining for HSD3B1 by adding 0.4 mM etiocholanolone as the steroid substrate and NAD^+^ as a cofactor and nitroblue as the staining solution (Payne et al., 1980). The purity of immature Leydig cells was >95%. Total three isolations were prepared.

### Culture of Immature Leydig Cells

Immature Leydig cells were seeded into the 6-well culture plates after isolation with a density of 5 × 10^5^ cells per well. Leydig cells were treated with 0, 10, and 100 nM TPT in 2.0 mL DMEM: F12 medium (Gibco, Grand Island, NY, United States) for 24 h. The concentrations were selected based on the previous study that showed TPT had a direct inhibition on steroidogenic enzyme 17β-hydroxysteroid dehydrogenase 3 activity from pig Leydig cells with IC_50_ of 48 nM (Ohno et al., 2005). Leydig cells were harvested for the analysis of reactive oxygen species (ROS) generation and apoptosis of Leydig cells.

### Measurement of Intracellular ROS Levels

Reactive oxygen species production was measured with the fluorescence dye 2′,7′-dichlorofluorescin diacetate (DCFH-DA) assay kit (Qcbio Science and Technologies Co., Shanghai, China) according to the manufacturer’s instruction. Briefly, isolated immature Leydig cells were plated into the 6-well plates at the density of 5 × 10^5^ cells per well and stabilized for 24 h. Then, cells were incubated with 0, 10, or 100 nM TPT for 24 h. Thereafter, cells were collected by digestion with trypsin, after which cells were suspended and incubated with 200 μL DCFH-DA for 20 min at 37°C in dark. Phosphate buffered saline (PBS) washing step was repeated and fluorescence intensity was determined by a DTX800 Multimode Detector (Beckman Coulter, Fullerton, CA, United States) with excitation of 485 nm and emission of 535 nm to determine ROS levels.

### Annexin V and PI Assay for Apoptosis

Immature Leydig cells freshly isolated as above were planted into a 6-well plate in a density of 5 × 10^5^ cells per well. Cells were incubated with 0, 10, or 100 nM TPT for 24 h. An Annexin V-FITC/PI Apoptosis Detection Kit (Nanjing KeyGEN Biotech, Nanjing, China) was used to evaluate both infant and terminal cellular apoptosis under the manufacturer’s instruction. Leydig cells were then collected by digestion with trypsin, washed with cold PBS, and resuspended in 200 μL Annexin V-binding buffer. Cells were further stained with FITC-labeled Annexin V and PI. Fluorescence of cells was detected by the Flow Cytometer as above.

### Statistical Analysis

Data are presented as the mean ± standard error (SEM). Statistical significance was analyzed by one-way ANOVA followed by *ad hoc* Turkey’s multiple comparison between two groups. Statistical analyses were performed using GraphPad Prism (version 6, GraphPad Software Inc., San Diego, CA, United States). Values were considered significant at *P* < 0.05.

## Results

### General Toxicological Parameters of TPT

Rat body weights were recorded during the course of TPT treatment and the testis and epididymal weights were recorded at the end of TPT treatment. TPT did not alter rat body weights, testis weights, and epididymal weights. Two rats at 2 mg/kg TPT group were dead at postnatal day 47. No other abnormal activities were observed during the course of TPT treatment (**Table 1**).

**Table 1:** General toxicological parameters after treatment of triphenyltin.

Parameters	Triphenyltin (TPT) dosage (mg/kg)				
	0	0.5	1	2
**Animal number**	**7**	**7**	**7**	**7**
**Body weight (g)**				
Before TPT	170.0 ± 5.2	176.3 ± 0.8	172.5 ± 11.2	166.6 ± 10.4
After TPT (PND52)	250.2 ± 6.5	249.2 ± 9.3	227.3 ± 13.6	212.4 ± 8.8
**Epididymis weight (g)**				
After TPT (PND52)	0.63 ± 0.01	0.68 ± 0.02	0.62 ± 0.01	0.60 ± 0.03
**Testes weight (g)**				
After TPT (PND52)	1.42 ± 0.06	1.45 ± 0.04	1.40 ± 0.03	1.38 ± 0.06

*PND, postnatal day. Mean ± SEM, n = 5–7. No significant difference between groups was observed.*

### TPT Reduces Serum Testosterone Levels

**FIGURE 1:**
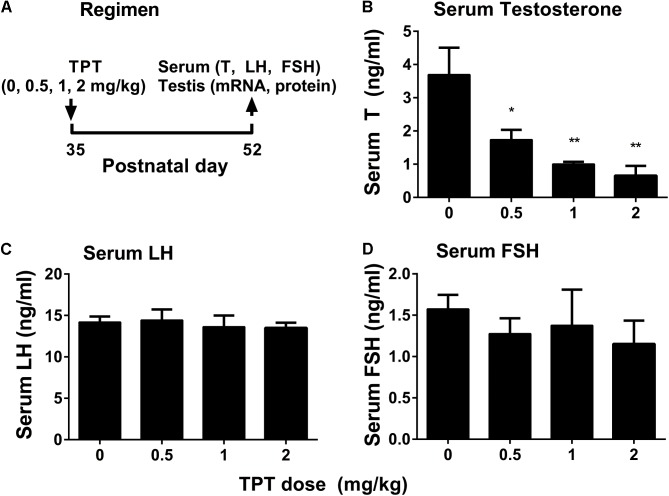
Regimen of TPT and serum testosterone, LH, and FSH levels after TPT treatment. **(A)** Regimen of TPT; **(B–D)** serum T, LH, and FSH levels, respectively. Mean ± SEM, *n* = 5–7; ^∗^*P* < 0.05 and ^∗∗^*P* < 0.01 indicate significant difference when compared to the control (0 mg/kg).

Triphenyltin chloride (0, 0.5, 1, and 2 mg/kg body weight/day) were gavaged to male rats starting from 35 days to 52 days postpartum (**Figure 1A**). TPT dose-dependently lowered serum testosterone levels (**Figure 1B**), being significant at 0.5 mg/kg and the higher doses. However, serum LH (**Figure 1C**) and FSH (**Figure 1D**) levels did not change when compared with the control. This indicated that TPT directly inhibited rat Leydig cell development.

### TPT Does Not Affect Leydig and Sertoli Cell Numbers

**FIGURE 2:**
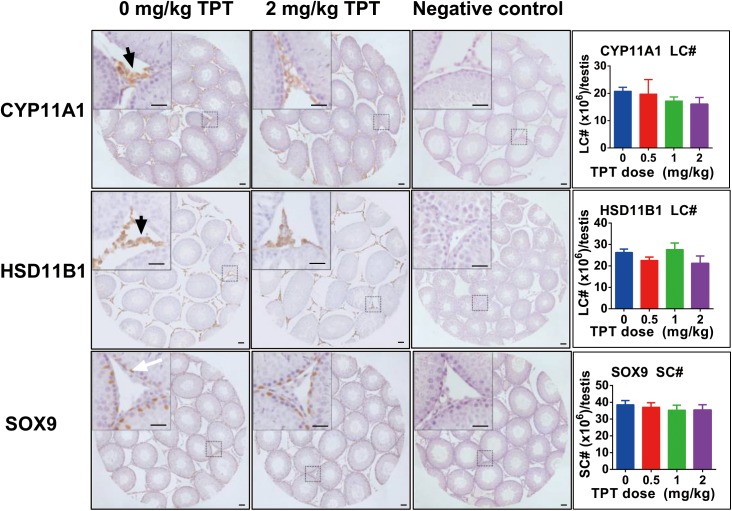
Immunohistochemical staining of CYP11A1, HSD11B1, and SOX9 in rat testis sections and the enumeration of Leydig and Sertoli cell numbers after TPT treatment. Representative images were used. Black arrow points to CYP11A1- or HSD11B1-positive Leydig cells. White arrow points to the SOX9-positive Sertoli cell. Bar = 30 μm. Mean ± SEM, *n* = 5–7. No significant difference between groups was observed.

CYP11A1 is a general biomarker for all Leydig cells in the Leydig cell lineage (Guo et al., 2013). HSD11B1 is a specific biomarker of Leydig cells at the advanced stage (immature and adult Leydig cells) (Phillips et al., 1989; Guo et al., 2013). We counted the cell number of CYP11A1-positive cells and HSD11B1-positive cells in the interstitium of the testis at the end of TPT treatment (**Figure 2**). It was found that TPT did not alter both CYP11A1-positive and HSD11B1-positive cell number, indicating that Leydig cell number was not altered after TPT treatment. We also stained Sertoli cells with the biomarker of SOX9 and found that the SOX9-positive cell number was not altered either by TPT, indicating that TPT did not affect Sertoli cell number (**Figure 2**).

### TPT Lowers Leydig Cell Size and Cytoplasmic Size

**FIGURE 3:**
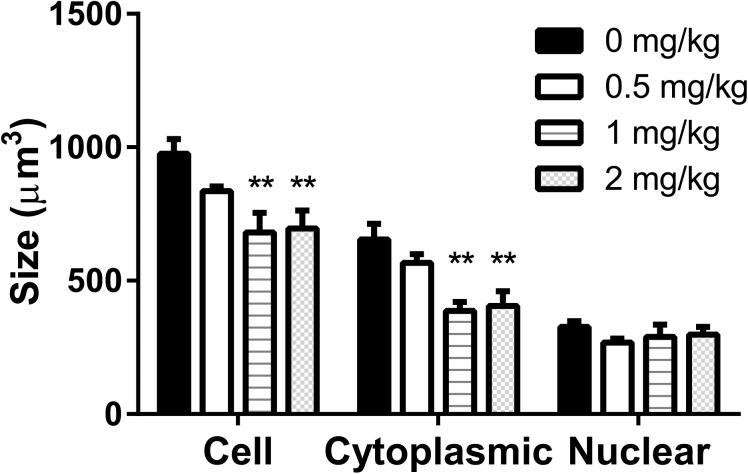
Leydig cell size, cytoplasmic size, and nuclear size in rat testis sections after TPT (0–2 mg/kg) treatment. Mean ± SEM, *n* = 5–7. ^∗∗^Indicates significant difference when compared to the control (TPT 0 mg/kg) at *P* < 0.01.

We further analyzed Leydig cell population by morphological measurement and found that TPT treatment decreased Leydig cell size and cytoplasmic size without having effects on the nuclear size (**Figure 3**). Usually, the more mature the Leydig cell is, the larger the cell is. This indicated that TPT might delay Leydig cell maturation process.

### TPT Downregulates Some Leydig Cell Gene Expression

**FIGURE 4:**
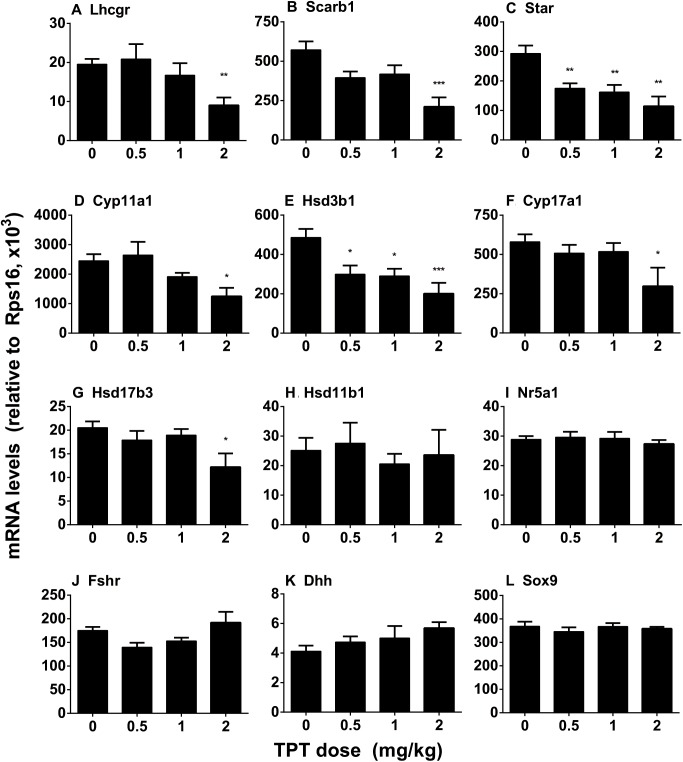
Expression levels of Leydig and Sertoli cell genes in the TPT-treated testis on the postnatal day 52. Leydig cell genes: *Lhcgr* **(A)**, *Scarb1* **(B)**, *Star* **(C)**, *Cyp11a1* **(D)**, *Hsd3b1* **(E)**, *Cyp17a1* **(F)**, *Hsd17b3* **(G)**, *Hsd11b1* **(H)**, and *Nr5a1* **(I)**. Sertoli cell genes: *Fshr* **(J)**, *Dhh* **(K)**, and *Sox9* **(L)**. Mean ± SEM, *n* = 5–7. Asterisks indicate significant difference when compared to the control (TBT 0 mg/kg) at ^∗^*P* < 0.05, ^∗∗^*P* < 0.01, and ^∗∗∗^*P* < 0.001, respectively.

The expression levels of Leydig (*Lhcgr*, *Scarb1*, *Star*, *Cyp11a1*, *Hsd3b1*, *Cyp17a1*, *Hsd17b3, Hsd11b1*, and *Nr5a1*) and Sertoli (*Fshr*, *Dhh*, and *Sox9*) cell genes were measured at the end of TPT treatment (**Figure 4**). TPT significantly downregulated the expression levels of *Star* and *Hsd3b1* at 0.5 mg/kg and the higher doses. It also significantly lowered the expression levels of *Lhcgr*, *Scarb1*, *Cyp11a1*, *Cyp17a1*, and *Hsd17b3* genes at 2 mg/kg dose. TPT did not affect *Hsd11b1*, *Nr5a1*, *Fshr*, *Dhh*, and *Sox9* gene expression levels. These results suggested that Leydig cells were more sensitive to TPT than Sertoli cells.

### TPT Decreases Some Leydig Cell Protein Levels

**FIGURE 5:**
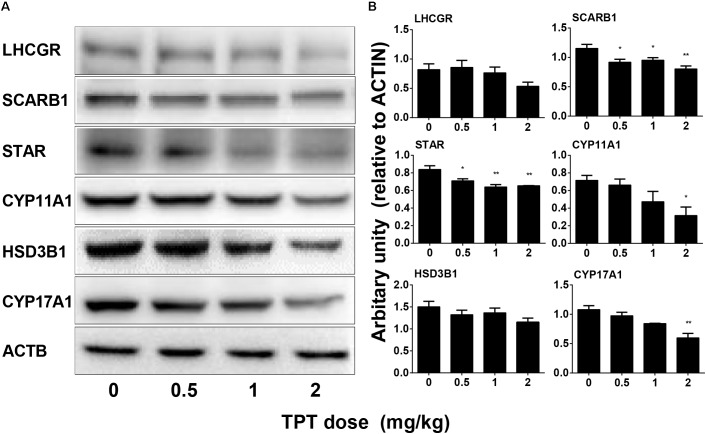
Protein levels of Leydig cell gene products in the TPT-treated testis on the postnatal day 52. **(A)** Gel images; **(B)** quantitative result. Leydig cell proteins: LHCGR, SCARB1, STAR, CYP11A1, HSD3B1, and CYP17A1. Mean ± SEM, *n* = 3. Asterisks indicate significant differences when compared to the control (TPT 0 mg/kg) at ^∗^*P* < 0.05, ^∗∗^*P* < 0.01, and ^∗∗∗^*P* < 0.001, respectively.

**FIGURE 6:**
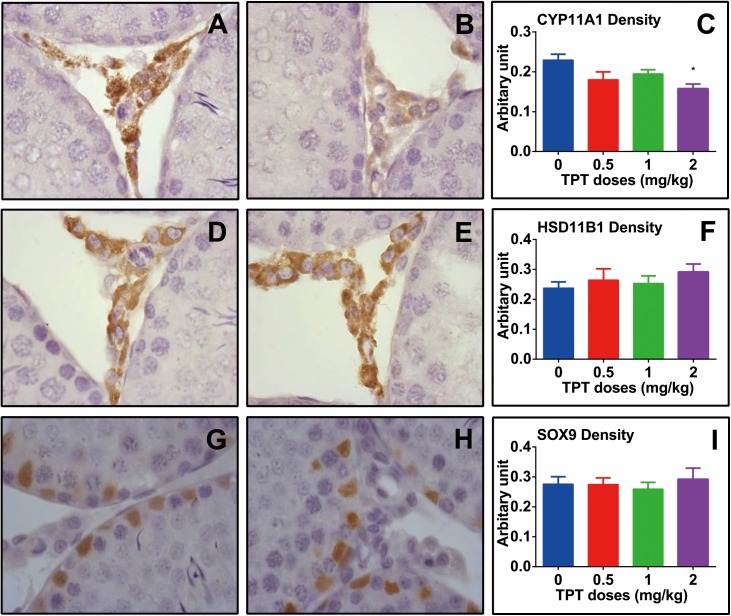
Semi-quantitative measurement of CYP11A1, HSD11B1, and SOX9 densities in rat testis after TPT treatment. **(A,B)** CYP11A1 staining; **(D,E)** HSD11B1 staining; **(G,H)** SOX9 staining; **(A,D,G)** control; **(B,E,H)** 2.0 mg/kg TPT. **(C,F,I)** Quantification of CYP11A1, HSD11B1, and SOX9 density, respectively. Mean ± SEM, *n* = 5–7. Asterisk indicates significant difference when compared to the control (TPT 0 mg/kg) at *^∗^P* < 0.05.

The levels of Leydig cell (LHCGR, SCARB1, STAR, CYP11A1, HSD3B1, CYP17A1, and HSD11B1) proteins were measured by Western blot. TPT lowered these protein levels in parallel with their mRNA expression levels (**Figure 5**). We further used a semi-quantitative measurement of CYP11A1 and HSD11B1 (Leydig cell proteins), as well as SOX9 (Sertoli cell protein) densities in the individual cell and found that TPT lowered CYP11A1 but not HSD11B1 and SOX9 densities (**Figure 6**). These results suggested that TPT impaired Leydig cell functions without affecting Sertoli cell functions at 2 mg/kg dose.

### Effects of TPT on Kinase Phosphorylation

**FIGURE 7:**
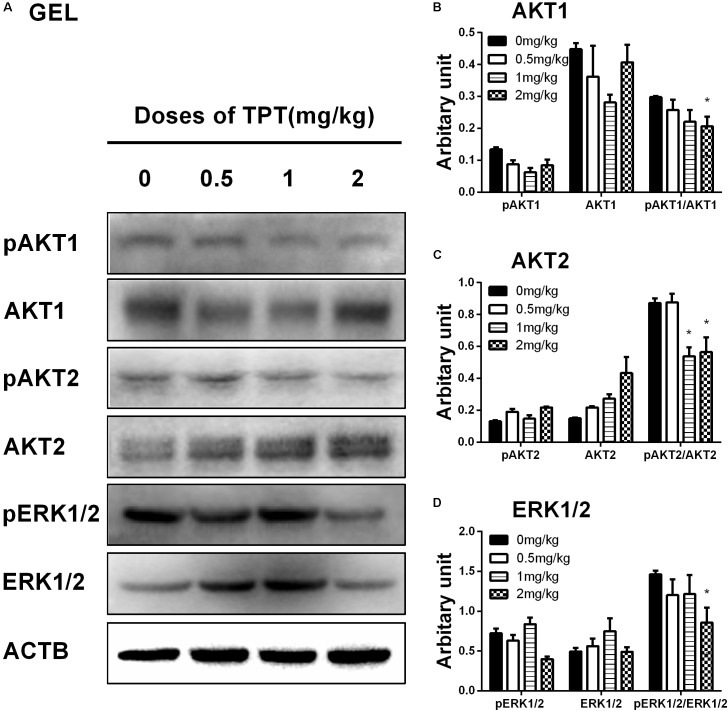
Protein levels of kinases and phosphorylated kinases in the TPT-treated testis on the postnatal day 52. **(A)** Gel images; **(B–D)** quantitative result. Proteins: AKT1 **(B)**, AKT2 **(C)**, and ERK1/2 **(D)**. Mean ± SEM, *n* = 5. Asterisk indicates significant difference when compared to the control (TPT 0 mg/kg) at ^∗^*P* < 0.05.

Many studies have demonstrated that AKT and ERK1/2 pathways participated in development of Leydig cells (Manna et al., 2006, 2007; Shiraishi and Ascoli, 2007). In the current study, we investigated the downstream signals after TPT treatment in the testis. TPT significantly decreased the ratios of pAKT1/AKT1 at 2 mg/kg and pAKT2/AKT2 at ≥1 mg/kg doses although it increased AKT2 at 2 mg/kg. TPT significantly decreased ERK1/2 and pERK1/2/ERK1/2 ratio (**Figure 7**). These results indicated that ERK1/2 and AKT pathways involved in the TPT-mediated suppression of Leydig cell development.

### TPT Increases ROS Production of Immature Leydig Cells *in vitro*

**FIGURE 8:**
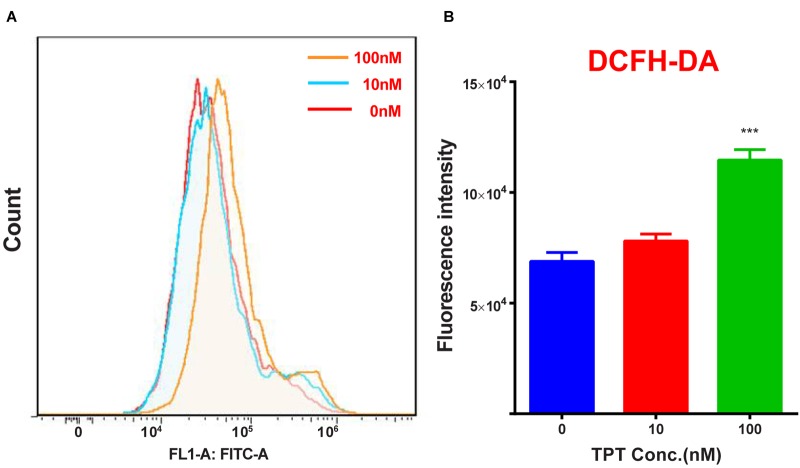
ROS production in immature Leydig cells after TPT treatment. **(A)** Count of ROS; **(B)**, quantitation data. Mean ± SEM, *n* = 3. Asterisk represents significant difference when compared to the control (0 nM TPT) at ^∗∗∗^*P* < 0.001.

As shown in **Figure 8**, there was a significant difference in ROS generation between control and TPT treatment group after exposure to 100 nM TPT for 24 h, indicating that at this concentration TPT could cause ROS generation.

### TPT Induces Apoptosis of Immature Leydig Cells *in vitro*

**FIGURE 9:**
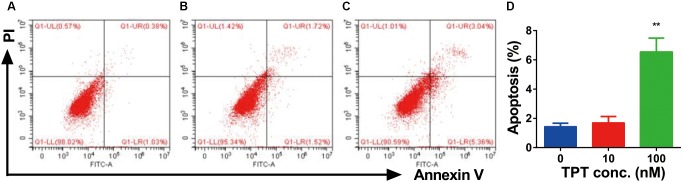
FACS analysis of Annexin V-FITC labeling of apoptotic Leydig cells treated with TPT *in vitro.* Cells in the Q1-LR quadrant were designated apoptotic (PI-negative/Annexin V-FITC-positive), cells in Q1-LL were designated living (PI-negative/Annexin V-FITC-negative), cells in Q1-UL were designated dead (PI-positive/Annexin V-FITC-positive), and cells in Q1-UR were designated damaged (PI-positive/Annexin V-FITC-negative). **(A–C)** The FACS spectrum after 0, 10, and 100 nM TPT treatment, respectively; **(D)** quantitative data, mean ± SEM, *n* = 3. Leydig cells treated with TPT (100 nM) for 24 h **(C)** showed increased frequencies of apoptotic labeling in the Q1-LR quadrant relative to untreated controls **(A)**. Asterisk indicates significant difference when compared to control (0 nM TPT) at ^∗∗^*P* < 0.01.

As shown in **Figure 9**, there was a significant difference in apoptosis rate between control and TPT treatment group after exposure to 100 nM TPT for 24 h, indicating that at this concentration TPT could cause apoptosis of Leydig cells.

## Discussion

The current study demonstrated that TPT significantly delayed the development of adult Leydig cells during puberty, thus lowering testosterone production. Apparently, TPT did not affect the Leydig cell number but reduced the steroidogenic capacity of the Leydig cell *per se*. TPT mainly downregulated many steroidogenic proteins, especially HSD3B1 and STAR.

The current study used 0.5, 1, and 2 mg/kg/day TPT. According to EPA reports (United States. Environmental Protection Agency. Prevention et al., 1999), no observed adverse effect level (NOAEL) and lowest observed adverse effect level (LOAEL) for reproductive and developmental toxicity (judged by the decreased litter, size, liver, and spleen weights) of TPT in rats were 0.25 and 0.925 mg/kg/day (accession number: 264667 to 264676), respectively, and the medium-term NOAEL/LOAEL (judged by the decreased body weight and gain and food consumption) for rats were <0.33 and 7.63 mg/kg/day (accession number: 00157771 and 261754), respectively. Apparently, 0.5 mg/kg/day did not cause any changes in body weights, testis weights, and epididymal weights of rats. The current study showed lower circulating testosterone levels, associated with unchanged FSH and LH levels in rats treated with TPT from 35 days to 52 days postpartum even at the dose of 0.5 mg/kg TPT, which did not cause overt toxicity. This indicated a possible inhibition of Leydig cell development. Indeed, Grote et al. (2004) examined 2, 6, or 12 mg/kg TPT on the male sexual development from 23 days postpartum for 30 days and found that the weights of reproductive organs were lower and testosterone levels were decreased in 6 and 12 mg TPT groups. Interestingly, when rats were exposed to 6 mg/kg TPT, serum LH levels were increased and this increase was also observed in adult mice, which had the elevated levels of serum LH and FSH levels with lowered testosterone levels after 1–2 mg/kg TPT treatment (Reddy et al., 2006). However, in the current study, we did not observe any alteration of serum LH and FSH levels after TPT treatment. This discrepancy could be due to the age difference during the TPT treatment.

Lower testosterone level was not contributed by the reduced Leydig cell number since both CYP11A1 and HSD11B1 Leydig cell number was not changed, but was contributed by the reduced capacity of androgen production of the Leydig cell *per se*. It was true that TPT treatment significantly downregulated many steroidogenic proteins including LHCGR, SCARB1, STAR, CYP11A1, HSD3B1, CYP17A1, and HSD17B3. The most sensitive responsive genes after TPT treatment were *Hsd3b1* and *Star*, which were significantly decreased at as low as 0.5 mg/kg TPT dose. *Star* encoded STAR protein, which was a rate-limiting step for cholesterol transportation from cytosol of Leydig cells into the inner membrane of the mitochondria for CYP11A1 catalysis (Manna et al., 2009). The serum testosterone levels were correlated to the levels of *Star* mRNA.

The mechanisms of TPT to inhibit androgen production could act at multiple levels. First, TPT could interfere with the transcription of steroidogenic protein-related genes. As shown in the present study, TPT downregulated many steroidogenic gene expression such as *Lhcgr*, *Scarb1*, *Star*, *Cyp11a1*, *Hsd3b1*, *Cyp17a1*, and *Hsd17b3*. A recent study using another organotin tributyltin also showed that the expression levels of SCARB1, CYP11A1, HSD3B1, CYP17A1, and HSD17B3 were downregulated in the adult male hamster testes after 65-day treatment (Kanimozhi et al., 2017). Secondly, TPT directly inhibited steroidogenic enzyme activities. Ohno et al. (2005) reported that TPT directly inhibited pig testis HSD17B3 with high potency (IC_50_ value of 48 nM) and P450 reductase with moderate potency (IC_50_ value of 22.8 μM). P450 reductase is an enzyme complex of CYP17A1 catalysis, at which it serves as its electron carrier. CYP17A1 catalysis requires 2 one-electron transfers from reduced nicotinamide adenine dinucleotide phosphate via P450 reductase, binding of substrate and oxygen, formation of the reactive heme-iron complex with oxygen, and finally substrate oxidation (Yoshimoto and Auchus, 2015). The mutation of P450 reductase could cause the impaired CYP17A1 activity (Fluck and Pandey, 2011).

We also examined the higher concentration (100 nM) of TPT on ROS generation and apoptosis *in vitro*. Indeed, this concentration increased ROS generation in rat immature Leydig cells. Redox imbalances are closely involved in TPT toxicity. Another organotin tributyltin has been shown to induce intracellular Ca^2+^ levels and this Ca^2+^ increase in Leydig cells might deplete anti-oxidant GSH (Mitra et al., 2014). Decreased GSH was followed by the elevation of ROS, which mediated lipid peroxidation (Cima and Ballarin, 2004) and had detrimental effects on critical components of the steroidogenic pathway, particularly the rate-limiting STAR (Diemer et al., 2003) and ROS increases could lead to the Leydig cell apoptosis (Guo et al., 2017). Indeed, other toxicants like the similar organotin tributyltin, nicotine from cigarettes, and perfluorooctane sulfonate from the environmental exposure increased ROS and induced Leydig cell apoptosis (Mitra et al., 2014; Zhang et al., 2014; Guo et al., 2017). However, this ROS pathway may not be the major one in TPT-mediated endocrine disrupting effects.

**FIGURE 10:**
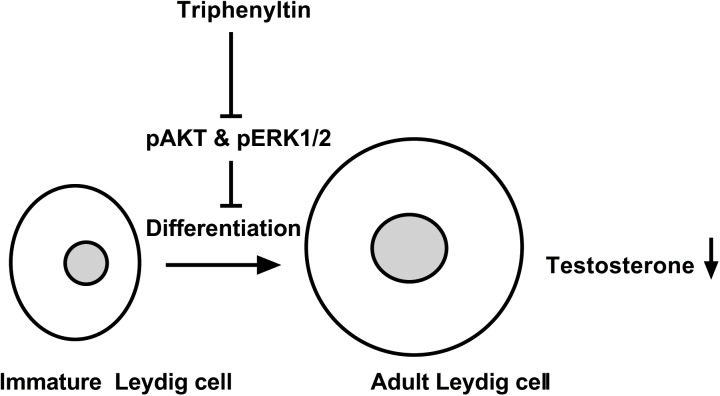
Illustration of the mechanism of TPT-mediated inhibition of testosterone production. TPT blocks the phosphorylation of AKT1, AKT2, and ERK1/2, thus delaying the differentiation of immature Leydig cells into adult Leydig cells and causing the decrease of testosterone production.

Many studies demonstrated that AKT and ERK1/2 signaling pathways took part in Leydig cell development (Manna et al., 2006, 2007; Renlund et al., 2006; Shiraishi and Ascoli, 2007). For example, epidermal growth factor, insulin-like growth factor 1, and LH can regulate AKT or ERK1/2 phosphorylation. AKT is an important regulator of Leydig cell development. It was found that AKT was mainly regulated by insulin-like growth factor 1 (Tai et al., 2009) and kit ligand (Rothschild et al., 2003). Indeed, previous studies showed that insulin-like growth factor 1 knockout in mice downregulated the expression of several Leydig cellg genes such as *Star*, *Cyp11a1*, *Hsd3b1*, and *Cyp17a1*, reduced Leydig cell number, and lowered testosterone biosynthesis (Baker et al., 1996; Hu et al., 2010). When kit ligand signaling was blocked, testosterone production in Leydig cells was also lowered (Rothschild et al., 2003). Insulin-like growth factor 1 and kit ligand mainly activated phosphatidylinositol 3-kinase (Rothschild et al., 2003) and this activation of could lead to increase in AKT phosphorylation (Tai et al., 2009; Zhou et al., 2016). Epidermal growth factor binds to its receptor caused activation of ERK1/2 in Leydig cells (Hirakawa and Ascoli, 2003; Shiraishi and Ascoli, 2007, 2008). LH after binding to LHCGR also activated ERK1/2 cascade in immature Leydig cells partially via epidermal growth factor receptor (Hirakawa and Ascoli, 2003; Shiraishi and Ascoli, 2007, 2008). Indeed, STAR is the substrate of ERK1/2 and phosphorylaton of ERK1/2 participates in cholesterol transportation (Poderoso et al., 2008). Although, we have not performed a complete characterization of the pathways by which the TPT enhances AKT and ERK1/2 phosphorylation, the current data presented demonstrated that AKT and ERK1/2 pathways were associated with Leydig cell development (**Figure 10**).

## Conclusion

We examined the effects of both *in vivo* and *in vitro* TPT exposure on pubertal Leydig cells and demonstrated that TPT was a potent inhibitor of Leydig cell development in rats. The negative effects of TPT seems most likely via directly disrupting many steroidogenic proteins, mainly STAR, thus leading to lower testosterone production, possibly via regulation of AKT and ERK1/2 phosphorylation.

## Author Contributions

XL and R-SG conceptualized the study design and analyzed the data. LL, LX, LM, YC, XwC, FG, TH, LC, TH, XfC, and QZ performed the experiments and collected the data. R-SG wrote the manuscript.

## Conflict of Interest Statement

The authors declare that the research was conducted in the absence of any commercial or financial relationships that could be construed as a potential conflict of interest.
