# Supplementary material for: Triphenyltin Chloride Delays Leydig Cell Maturation During Puberty in Rats
Source: Front Pharmacol. 2018 Aug 10;9:833. doi: 10.3389/fphar.2018.00833 (PMC6095986; doi:10.3389/fphar.2018.00833)
Supplement: Supplementary file 1 [file Table_1.DOC]

**Supplementary Table 1**. Primer information

| **Primer**  **Symbol** | **Gene name** | **Primer direction** | **Sequences (5’to 3’)** | **PCR**  **(bp)** | **Accession** |
| --- | --- | --- | --- | --- | --- |
| Lhcgr | Luteinizing hormone receptor | Forward | CTGCGCTGTCCTGGCC | 103 | NM_012978 |
|  |  | Reverse | CGACCTCATTAAGTCCCCTGAA |  |  |
| Scarb1 | Scavenger receptor class B, member 1 | Forward | ATGGTACTGCCGGGCAGAT | 117 | NM_031541 |
|  |  | Reverse | CGAACACCCTTGATTCCTGGTA |  |  |
| Star | Steroidogenic acute regulatory protein | Forward | CCCAAATGTCAAGGAAATCA | 187 | NM_031558 |
|  |  | Reverse | AGGCATCTCCCCAAAGTG |  |  |
| Cyp11a1 | Cholesterol side chain cleavage enzyme | Forward | AAGTATCCGTGATGTGGG | 127 | NM_017286 |
|  |  | Reverse | TCATACAGTGTCGCCTTTTCT |  |  |
| Hsd3b1 | 3β-Hydroxysteroid dehydrogenase 1 | Forward | CCCTGCTCTACTGGCTTGC | 189 | NM_001007719 |
|  |  | Reverse | TCTGCTTGGCTTCCTCCC |  |  |
| Cyp17a1 | 17α-hydroxylase/ 17,20-lyase | Forward | TGGCTTTCCTGGTGCACAATC | 90 | NM_012753 |
|  |  | Reverse | TGAAAGTTGGTGTTCGGCTGAAG |  |  |
| Hsd17b3 | 17β-Hydroxysteroid dehydrogenase 3 | Forward | TGAAAGTTGGTGTTCGGCTGAAG | 202 | NM_054007 |
|  |  | Reverse | TGAAAGTTGGTGTTCGGCTGAAG |  |  |
| Hsd11b1 | [11β-Hydroxysteroid dehydrogenase 1](https://www.baidu.com/link?url=RASn5FVJOHQO5F8yLZuLK2GaE-txBvaDG-Aix0zS1TOK-H6BDM3SQ-dtmZqJCiib&wd=&eqid=adb6e42d00037867000000045854b8a0) | Forward | GAAGAAGCATGGAGGTCA | 290 | NM_017080 |
|  |  | Reverse | CTCAAGATTATCCCAGAGG |  |  |
| Nr5a1 | Nuclear receptor  steroidogenic factor 1 | Forward | CAGAGCTGCAAAATCGACAA | 186 | NM_053344 |
|  |  | Reverse | CCCGAATCTGTGCTTTCTTC |  |  |
| Sox9 | Sox9 gene | Forward | GCAGCGTGGGGTTGTg | 172 | NM_138547 |
|  |  | Reverse | TGGATGATTGGGATGGTCA |  |  |
| Dhh | Dessert Hedgehog | Forward | AACCCCGACATAATCTTCA | 150 | NM_053367 |
|  |  | Reverse | CTCGTCCCAACCTTCAGT |  |  |
| Fshr | Follicle stimulating hormone receptor | Forward | CCACAAGCCAATACAAACTAACT | 327 | NM_199237 |
|  |  | Reverse | CAAAAGTCCAGCCCAATACC |  |  |
| Rps16 | Ribosomal protein | Forward | AAGTCTTCGGACGCAAGAAA | 148 | [NM_001169146](https://www.ncbi.nlm.nih.gov/entrez/viewer.fcgi?db=nucleotide&id=310703681) |
|  | S16 | Reverse | TTGCCCAGAAGCAGAACAG |  |  |
