# Supplementary material for: Triphenyltin Chloride Delays Leydig Cell Maturation During Puberty in Rats
Source: Front Pharmacol. 2018 Aug 10;9:833. doi: 10.3389/fphar.2018.00833 (PMC6095986; doi:10.3389/fphar.2018.00833)
Supplement: Supplementary file 2 [file Table_2.docx]

**Supplementary Table S2. Antibodies**

| **Antibody** | **Species** | **Vendor (City, State, catalogue)** | **Dilution** | |
| --- | --- | --- | --- | --- |
|  |  |  | **WB** | **HS** |
| ACTB | rabbit | Cell Signaling Technology (Danvers, MA) | 1:1000 | ND |
| NR5A1 | rabbit | Abcam (San Francisco, CA) | 1:1000 | ND |
| LHCGR | rabbit | Multi Sciences (Hangzhou, China) | 1:1000 | ND |
| STAR | rabbit | Cell Signaling Technology (Danvers, MA) | 1:1000 | ND |
| SCARB1 | rabbit | Multi Sciences (Hangzhou, China) | 1:1000 | ND |
| CYP11A1 | rabbit | Cell Signaling Technology (Danvers, MA) | 1:1000 | 1:500 |
| CYP17A1 | rabbit | Abcam (San Francisco, CA) | 1:1000 | ND |
| HSD11B1 | rabbit | Abcam (San Francisco, CA) | 1:1000 | 1: 500 |
| HSD3B1 | rabbit | Abcam (San Francisco, CA) | 1:1000 | ND |

ND = Not detected; WB = Western blot; HS = Histochemical staining.
